# The Role of Tumour Metabolism in Cisplatin Resistance

**DOI:** 10.3389/fmolb.2021.691795

**Published:** 2021-06-23

**Authors:** Lude Wang, Xiaoya Zhao, Jianfei Fu, Wenxia Xu, Jianlie Yuan

**Affiliations:** ^1^ Central Laboratory, Affiliated Jinhua Hospital, Zhejiang University School of Medicine, Jinhua, China; ^2^ Department of Medical Oncology, Affiliated Jinhua Hospital, Zhejiang University School of Medicine, Jinhua, China; ^3^ Department of Neurosurgery, Affiliated Jinhua Hospital, Zhejiang University School of Medicine, Jinhua, China

**Keywords:** tumour metabolism, cisplatin, resistance, DNA damage repair, ROS

## Abstract

Cisplatin is a chemotherapy drug commonly used in cancer treatment. Tumour cells are more sensitive to cisplatin than normal cells. Cisplatin exerts an antitumour effect by interfering with DNA replication and transcription processes. However, the drug-resistance properties of tumour cells often cause loss of cisplatin efficacy and failure of chemotherapy, leading to tumour progression. Owing to the large amounts of energy and compounds required by tumour cells, metabolic reprogramming plays an important part in the occurrence and development of tumours. The interplay between DNA damage repair and metabolism also has an effect on cisplatin resistance; the molecular changes to glucose metabolism, amino acid metabolism, lipid metabolism, and other metabolic pathways affect the cisplatin resistance of tumour cells. Here, we review the mechanism of action of cisplatin, the mechanism of resistance to cisplatin, the role of metabolic remodelling in tumorigenesis and development, and the effects of common metabolic pathways on cisplatin resistance.

## Introduction

Cancers are systemic and complex diseases that seriously endanger human health, and their incidence is increasing because of the influence of environmental pollution and modern living habits. Current treatments for tumours include surgery, chemotherapy, radiotherapy, targeted therapy, and immunotherapy (McCarthy, 2006; Englinger et al., 2019). In recent years, molecular-mechanism-based targeted therapies and immunotherapy have shown great progress. However, the targeted therapy approach is limited by the low frequencies of gene mutations and small experimental population sizes. The use of immunotherapy also encounters many challenges, with few definitive answers on how to select the population likely to benefit, optimize the immunotherapy regimen, evaluate the effects of immunotherapy, and solve drug resistance and other urgent problems (Nishino et al., 2013; Carbone et al., 2017; Sharma et al., 2017). Chemotherapy remains the most widely used adjuvant treatment for cancer. Platinum drugs, including cisplatin, carboplatin, and oxaliplatin, are commonly used for most tumour types. Their basic pharmacological mechanism involves internal and interchain crosslinks created by binding to DNA, inhibition of DNA replication and transcription, and then induction of damage to double-stranded DNA. Cisplatin, a first-generation platinum drug, is used as a first-line therapy in clinical practice and has a good inhibitory effect on solid tumours including testicular cancer, ovarian cancer, lung cancer, stomach cancer, head and neck tumours, cervical cancer, and breast cancer (Dasari and Tchounwou, 2014). However, it is associated with numerous undesirable side effects including severe kidney problems, allergic reactions, decreased immunity to infections, gastrointestinal disorders, haemorrhage, and hearing loss especially in younger patients (Dasari and Tchounwou, 2014). Moreover, drug resistance of tumour cells often leads to loss of cisplatin efficacy and failure of chemotherapy, resulting in tumour progression (Q. Wu et al., 2014; Cree and Charlton, 2017). Previous in-depth studies on cisplatin resistance have addressed the contribution of genetics, epigenetics, and signal transduction pathways. Increasing attention has been given to the role of tumour metabolism in cisplatin resistance.

Tumour tissue is composed of cancer cells with different genetic and/or epigenetic backgrounds and surrounding stromal cells, a condition known as intra-tumoral heterogeneity (Hanahan and Weinberg, 2011; L. V. Nguyen et al., 2012). The microenvironment around cancer cells is completely different from that around normal cells. Therefore, tumour cells must demonstrate a rapid adaptive response to hypoxia and hypotrophic conditions. This phenomenon of bioenergetics in tumour cells, known as “metabolic reprogramming” (Yoshida, 2015), has been identified as one of the 10 characteristics of cancer. Remodelling of glucose, amino acid, and lipid metabolism is an important factor for promoting tumour development (Biswas, 2015). Metabolic reprogramming, on the one hand, meets the energy and material requirements of tumours; on the other hand, it involves epigenetic regulation, thereby playing an important part in tumour formation, metastasis, drug resistance, and other processes (Biswas, 2015; Wettersten et al., 2017). The processes by which cisplatin induces tumour cell death and by which tumour cells resist cisplatin-induced death are accompanied by metabolic reprogramming. Targeting metabolic processes therefore represents a potential novel strategy to reverse cisplatin resistance. The role of metabolic reprogramming in cisplatin resistance is reviewed in this paper.

## Discovery and Mechanism of Cisplatin Action

The antitumour effect of cisplatin was discovered in the 1960s by American physicist B. Rosenberg (Rosenberg et al., 1965), who associated the shapes of cell mitotic filaments with an electric or magnetic dipole field direction map and studied the effects of electric fields on bacterial division to discover disinfectants. In the course of this research, he found that when the platinum electrode was energized, *Escherichia coli* cells were 300-fold longer than the corresponding non-energized normal cells (Muggia et al., 2015; Rancoule et al., 2017). To study this phenomenon, T. Krigas tested the bacteria with all the products isolated from the culture and eventually found that [Pt (Ⅳ) (NH_3_)_2_Cl_4_] was a platinum-activated complex produced by electrolysis in nutrient solution that contributed to *E. coli* filamentation (Rosenberg et al., 1967). Krigas then asked, “Does only tetravalent platinum have this activity?” To answer this question, Krigas synthesized divalent platinum [Pt (Ⅱ) (NH_3_)_2_Cl_4_] and found that its activity was stronger than that of tetravalent platinum. [Pt (Ⅱ) (NH_3_)_2_Cl_4_] has both cis- and trans-structures, and its cis-structures have active trans-structures; the cis-structure is cisplatin (ROSENBERG et al., 1965). Rosenberg then applied cisplatin in antitumour research and found that it had good antitumour activity (Rosenberg et al., 1967). In 1978, the United States Food and Drug Administration approved cisplatin as a new anticancer drug, ushering in a new era of platinum drug development and applications (Prestayko et al., 1979). Later, a large number of studies demonstrated the antitumour effects of cisplatin on solid tumours including ovarian cancer, testicular cancer, and head and neck tumours (Kaye et al., 1992; Tanaka et al., 2018; de Vries et al., 2020).

Following the discovery of the anticancer effect of cisplatin, its mechanism of action was studied. The biochemical mechanism by which cisplatin crosses the cell membrane is still not completely understood. Recently, many studies have demonstrated that cisplatin enters cells through the copper trafficking system, which includes members of the copper transport (Ctr) protein family such as Ctr1 and Ctr2 (Kilari et al., 2016). The ATP7A and ATP7B copper pumps are also associated with the pumping of cisplatin. Cisplatin is inert and must be intracellularly activated by a series of aquation reactions that consist of the substitution of one or both cis-chloro groups with water molecules. Therefore, the activation of cisplatin depends on environmental conditions. In the blood or extracellular tissue fluid, the physiological chloride concentration is approximately 100 mmol/L, and the activity of cisplatin is low. Inside cells, the concentration of chloride ions decreases to only a few mmol/L, enabling the generation of highly reactive mono-and bi-aquated cisplatin forms (Chu et al., 1994).

The anticancer mechanism of cisplatin can be divided into nuclear and cytoplasmic modules according to localization. Aquated cisplatin avidly binds DNA, with a predilection for nucleophilic N7-sites on purine bases, resulting in 1,2- or 1,3-intrastrand crosslinks (P. Chen et al., 2016) and a lower percentage of interstrand crosslinks (Ming et al., 2017). These interactions lead to damage to the DNA double helix structure and interfere with replication and transcription. In particular 1,2-intrastrand ApG and CpG crosslinks have been identified as the most prominent cisplatin-induced DNA lesions and have been suggested to account for most, if not all, cisplatin cytotoxicity. In addition, the altered structure of the DNA makes it unrecognisable by DNA-damage-repair proteins. In general, the main roles of cisplatin in the nucleus are to inhibit DNA replication and RNA transcription, arrest the cell cycle, and cause programmed cell death. However, only ∼1% of intracellular cisplatin binds to nuclear DNA (Gonzalez et al., 2001), and cisplatin has been shown to have significant cytotoxicity to enucleated cells (Berndtsson et al., 2007). The larger proportion of intracellular cisplatin interacts with cytoplasm nucleophiles such as glutathione (GSH), mitochondrial DNA, proteins, phospholipids and phosphatidylserine in membranes, sulphur donors, and other mitochondrial structures (Galluzzi et al., 2014). These interactions result in reduced isotonic consumption and/or direct maintenance of reactive oxygen species (ROS) production. ROS have a dual role in cisplatin cytotoxicity, directly triggering mitochondrial outer membrane permeabilization (MOMP) or aggravating DNA damage induced by cisplatin (Sancho-Martinez et al., 2012).

**FIGURE 1:**
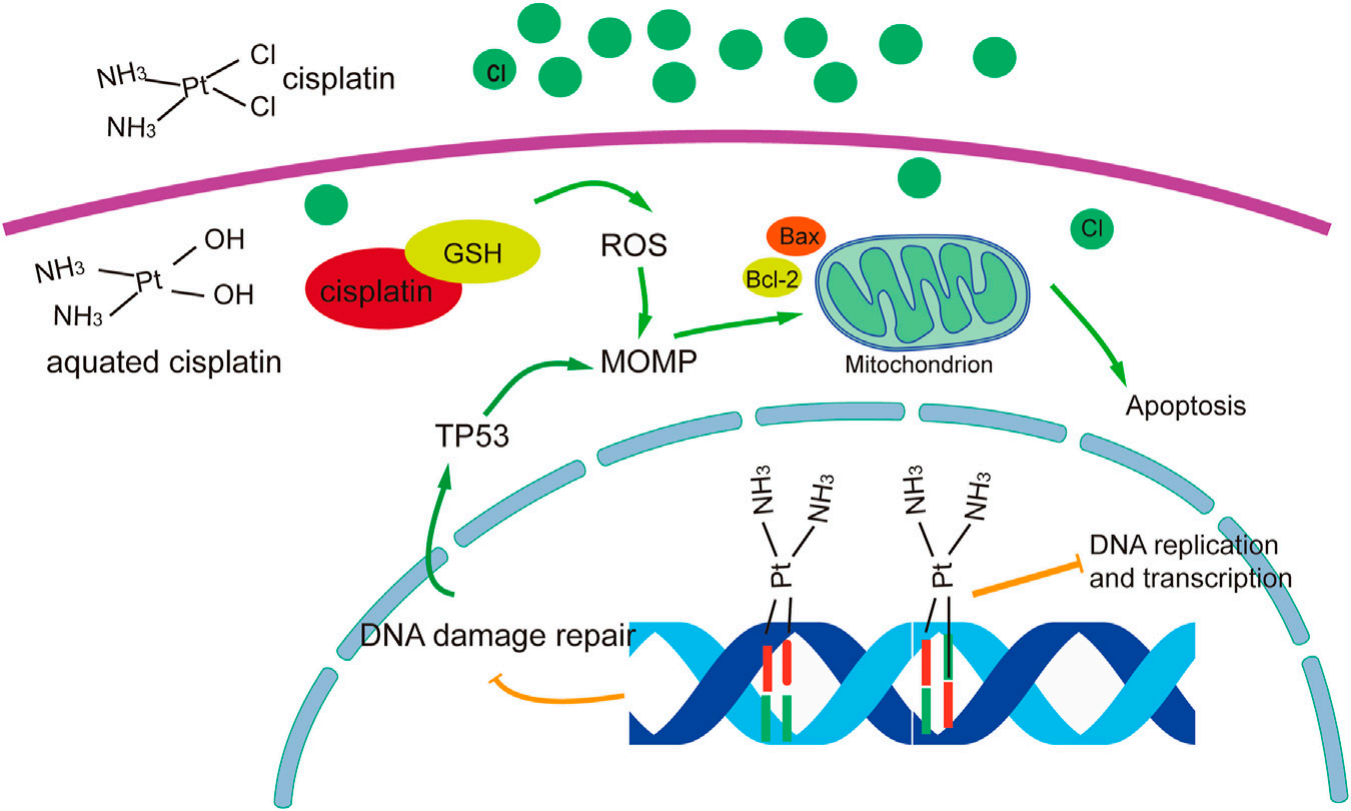
Overview of molecular mechanisms of cisplatin in cancer treatment. The figure was drawn in adobe illustrator.

The most effective mode of action of cisplatin involves the DNA damage response and mitochondrial apoptosis. Cisplatin-induced lesions cause distortions in DNA that can be identified by multiple repair pathways. Among these, the nucleotide excision repair (NER) and mismatch repair (MMR) systems are the most prominent mechanisms for the removal of cisplatin. If the damage cannot be repaired, cells become committed to (usually apoptotic) death. This involves the sequential activation of the ATR (ataxia telangiectasia mutated and RAD3-related protein, a sensor of DNA damage) and checkpoint kinase 1 (the most prominent substrate and downstream effector of ATR), which in turn phosphorylates the tumour suppression protein TP53. TP53 activates several genes whose products promote MOMP, thereby triggering endogenous apoptosis, as well as genes encoding components of exogenous apoptotic pathways. The extrinsic pathway is activated when the ligand binds to members of the tumour necrosis factor-α receptor superfamily and then forms the death-inducing signalling complex through oligomerization of the connector molecule and recruitment of procaspase-8 (Kischkel et al., 1995). The intrinsic pathways are initiated by cellular stresses such as DNA damage, leading to the release of cytochrome C by mitochondria, which activates procaspase-9. Bcl-2 family proteins regulate DNA-damage-induced apoptosis by regulating the release of mitochondrial cytochrome C in response to DNA damage (Nunez et al., 1998) (Figure 1).

## Clinical Applications of Cisplatin in Tumour Treatment

Cisplatin is widely used in the treatment of various cancers owing to its excellent anticancer effects (Table 1). Induction chemotherapy followed by radiation therapy (RT) is an organ-sparing treatment approach targeted to selected sub-sites of locally advanced head and neck squamous cell carcinoma. Induction regimens originally included cisplatin and 5-fluorouracil (5-FU) (PF) (Forastiere et al., 2003). More recent phase III trials have shown that the addition of docetaxel to PF results in superior efficacy in patients treated with RT (Vermorken et al., 2007) or carboplatin and RT (Posner et al., 2007). The standard chemotherapy for the initial treatment of ovarian cancer is a combination of a platinum analogue with paclitaxel (McGuire et al., 1996; Ozols et al., 2003); Deborah K found that intravenous paclitaxel plus intraperitoneal cisplatin and paclitaxel improved survival in patients with optimally debulked stage III ovarian cancer compared with intravenous paclitaxel plus cisplatin (Armstrong et al., 2006). In 1993, Housset and colleagues reported encouraging results with regard to bladder preservation and patient compliance with a hypofractionated twice-a-day radiation approach employing concurrent cisplatin and 5-FU that could be safely given as an outpatient regimen (Housset et al., 1993). Treatment of TC depends on stage and tumour type, i.e., seminoma or non-seminoma. Patients with disseminated non-seminoma (intermediate or low risk IGCCC) are treated with four courses of the BEP (bleomycin, etoposide and cisplatin) or VIP (etoposide, ifosfamide and cisplatin) regimen after surgical removal of the affected testicle. In cases of residual disease after completion of chemotherapy, patients undergo surgical removal of affected lymph nodes and/or metastases that had not completely disappeared after chemotherapy. Approximately 10–15% of patients with disseminated disease will need second-line treatment as a consequence of relapse or refractory disease (Adra and Einhorn, 2017). Various effective salvage strategies are currently available. The choice of standard-dose salvage treatment depends on which drugs were initially used in combination with cisplatin. Some common and effective standard-dose salvage treatments have been reported, with long-term remission rates ranging from 23 to 54% using VIP (McCaffrey et al., 1997; Miller et al., 1997), 63% using TIP (paclitaxel, ifosfamide and cisplatin) (Kondagunta et al., 2005), 24% using VeIP (vinblastine, ifosfamide, and cisplatin (Loehrer et al., 1998), and 51% using GIP (gemcitabine, ifosfamide and cisplatin) (Fizazi et al., 2014). In patients with advanced non-small-cell lung cancer, cisplatin plus etoposide was more effective but also more toxic than carboplatin plus etoposide (Klastersky et al., 1990; Faivre-Finn et al., 2017). Patients with limited-stage small-cell lung cancer were treated with concurrent twice-daily chest radiotherapy and etoposide/cisplatin followed by cyclophosphamide, doxorubicin, and vincristine (Johnson et al., 1996 Mar). Compared with paclitaxel plus cisplatin, paclitaxel plus carboplatin is not inferior and should be a standard treatment option for metastatic or recurrent cervical cancer; however, cisplatin is still the key drug for patients who have not received platinum agents (Kitagawa et al., 2015). 5- Fluorouracil combining with cisplatin (FP), capecitabine plus cisplatin (XP) regimen, epirubicin/cisplatin–5-FU (ECF), as well as 5-FU, an anthracycline and cisplatinis are adopted as standard reference regimens for patients with gastric cancer (Kang et al., 2009; Lee et al., 2012). Brandes et al. found that cisplatin plus temozolomide appeared to be effective in chemotherapy-naive patients with recurrent glioblastoma multiforme, with an acceptable level of toxicity (Brandes et al., 2004). The CBCSG006 trial reported the superior efficacy of a cisplatin plus gemcitabine (GP) regimen compared with paclitaxel plus gemcitabine as a first-line treatment for metastatic triple-negative breast cancer (Hu et al., 2015; J. Zhang et al., 2018a).

**TABLE 1:** The chemotherapy regimens of cisplatin in various tumours.

Tumour	Cisplatin chemotherapy regimens
Head and neck squamous cell carcinoma	cisplatin and 5-fluorouracil Armstrong et al. (2006); Kelland, (2007)
Ovarian cancer	cisplatin and paclitaxel McGuire et al. (1996); Ozols et al. (2003)
Bladder cancer	cisplatin and 5-fluorouracil Kaufman et al. (2000)
Testicular cancer	cisplatin, ifosfamide and etoposide Housset et al. (1993)
Lung cancer	cisplatin and etoposide Klastersky et al. (1990); Faivre-Finn et al. (2017)
Cervical cancer	cisplatin and paclitaxel Kitagawa et al. (2015)
Stomach cancer	cisplatin and capecitabine Lee et al. (2012)
Glioblastoma cancer	cisplatin and temozolomide Brandes et al. (2004)
Breast cancer	cisplatin and gemcitabine Hu et al. (2015); Zhang et al. (2018a)

## Molecular Mechanism of Cisplatin Resistance

Despite the successful application of cisplatin treatment against several cancer types, the effectiveness of the therapy is often limited by resistance, leading to therapeutic failure. DNA-damage-mediated apoptotic signals can be attenuated, and the resistance that ensues is a major limitation of cisplatin-based chemotherapy. Drug resistance is still the major obstacle to successful chemotherapy. Given the multiple mechanisms of cytotoxicity exerted by cisplatin, the cisplatin-resistant phenotype of cancer can be due to alterations in one or more of these molecular circuits (Galluzzi et al., 2012). The mechanism of cisplatin resistance includes the following main aspects.

**FIGURE 2:**
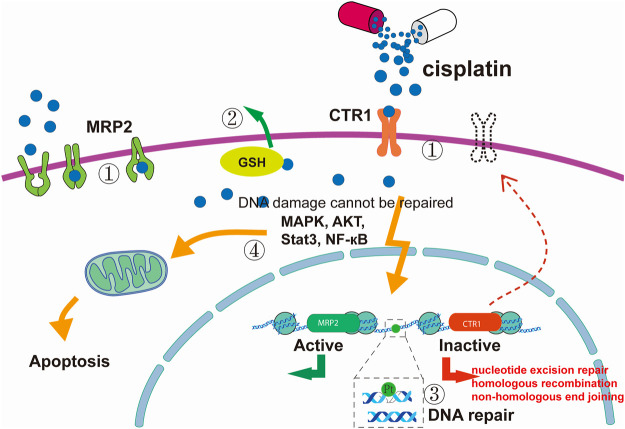
Cisplatin resistance mechanism. (1) Reduced intracellular accumulation of cisplatin. (2) Increased sequestration of cisplatin by GSH and other cytoplasmic scavengers with nucleophilic properties. (3) Enhanced DNA damage repair ability. (4) Defects in apoptotic signal transduction pathways. The figure was drawn in Adobe Illustrator.

1) Decreased drug uptake or increased drug effusion. This can significantly reduce cisplatin adducts, resulting in reduced toxicity and resistance. Ctr1 is a transmembrane protein with an important role in the cellular uptake of cisplatin. Studies have shown that cisplatin at clinical concentrations reduces the expression of CTR1, leading to a reduction in cisplatin uptake (Holzer et al., 2006). Overexpression of the ABC family transporter MRP2 leads to cisplatin being pumped from the cell into the extracellular space, reducing the intracellular cisplatin concentration (Borst et al., 2000; Liedert et al., 2003).
2) Increased sequestration of cisplatin by GSH and other cytoplasmic scavengers with nucleophilic properties. Aquated cisplatin binds to cytoplasmic nucleophilic substances, including GSH, methionine, metallothionein, and other cysteine-rich proteins. The activation of GSH detoxification and metallothionein systems by nucleophilic substances serving as cytoplasmic scavenging agents also accelerates the removal of cisplatin from cells (Mandic et al., 2003).
3) The sensitivity of tumour cells to cisplatin is related to the molecular damage caused by the direct binding of cisplatin to its target. Once cisplatin reacts with DNA, the cell must clear or tolerate the lesions to survive. Thus, cisplatin resistance is also related to DNA damage repair ability. The repair of damage to double-stranded DNA caused by cross-linking requires the joint participation of different repair mechanisms. Previous studies have found that NER (Shuck et al., 2008), homologous recombination (Telli et al., 2016), non-homologous end joining (Diggle et al., 2005; Sears and Turchi, 2012), and other repair mechanisms are involved.
4) The continuous action of cisplatin leads to abnormalities in the signal regulation networks in tumour cells, resulting in strong anti-apoptosis ability and resistance of cells to cisplatin. For example, TP53 is inactivated in approximately one-half of human tumours, endowing tumour cells with anti-apoptotic ability (Vousden and Lane, 2007). Other studies have found that the MAPK pathway (which has a critical role in regulating cisplatin-induced apoptosis) cannot be activated in cisplatin-tolerant cells, and, as a result, the FAS/FASL system (an inducer of extrinsic apoptosis) cannot be activated to enable cell survival (Spierings et al., 2003). In addition, PI3K/AKT, NF-κB, Stat3, and other signalling pathways are also involved in the regulation of cisplatin resistance (Mitsuuchi et al., 2000) (Figure 2).

These resistance mechanisms explain the changes in the genome, proteome, and signal transduction pathways that occur under the action of cisplatin. This does not fully explain the resistance mechanism of cisplatin. New research fields such as metabolic reprogramming are gradually being developed; we will elaborate on these in detail below.

## The Metabolic Remodelling Process of Tumours

In recent years, the understanding of malignant tumours has gradually changed from the concept of a “genetic disease” to one of a “metabolic disease” (Wishart, 2015), and metabolic remodelling has been recognized as one of the ten characteristics of tumours. Obesity, diabetes, dyslipidaemia, and other metabolic diseases are related to the development of tumours. Metabolic changes create selective advantages for tumour growth, proliferation, and survival. Metabolic processes produce energy and anabolic growth substrates to sustain cell survival and proliferation. Tumour cells meet the energy, biosynthesis, and oxidation-reduction reaction requirements for rapid and continuous proliferation through metabolic remodelling, which involves glycometabolism, amino acid metabolism, lipid metabolism, and other processes (Mathieu and Ruohola-Baker, 2017). Normal cells dissipate glucose energy mainly through the glycolytic–tricarboxylic acid (TCA) cycle–phosphorylation pathway under aerobic conditions; however, owing to their higher demand for energy, tumour cells rely on glycolysis as the main pathway of energy production.

This remodelling of glucose metabolism in tumour cells is known as the Warburg effect (Warburg et al., 1927) and also involves changes in the metabolic intermediates that provide biosynthetic materials for the rapid growth and division of tumour cells. These changes in metabolite levels vary among different cancers; for instance, a high-glycine diet may prevent breast cancer (Y. Wang et al., 2018b), serine metabolism is dysregulated in many tumours (Mattaini et al., 2016), asparaginase is an integral component of multiagent chemotherapy regimens for the treatment of acute lymphoblastic leukaemia (Salzer et al., 2018), histone hypermethylation can be induced in V600EBRAF melanoma cells by withdrawing glutamine (Cluntun et al., 2017), and high lactate levels are predictive of metastasis and restricted patient survival (Choi et al., 2013). Cellular glucose metabolism and cancer metabolism in general were not previously considered to be major branches of cancer biology, and high cellular glucose metabolism has only recently been recognized as one of the hallmarks of cancer by biologists (Hay, 2016). Lipid metabolism remodelling has an important role in the occurrence and development of hepatocellular carcinoma (HCC), mainly involving lipid biosynthesis and desaturation caused by upregulation of numerous crucial enzymes [ACL, acetyl-CoA carboxylase (ACC), fatty acid synthase (FASN), and stearoyl-coenzyme A desaturase-1 (SCD1)] in fatty acid biosynthesis. Monosaturated fatty acids, produced from saturated fatty acids by SCD1, are vital for membrane synthesis and prostaglandin synthesis and serve as sources for triacylglycerols. They influence cancer cell survival by contributing to autophagy activation, promoting cell membrane turnover, influencing intracellular signalling and gene transcription, and enhancing energy production (Pope et al., 2019). The three major metabolisms are not only independent of each other but are also related to each other through the TCA cycle. Below we will introduce the link between metabolic reprogramming and cisplatin resistance.

## Roles of Metabolic Processes in DNA Damage Repair

As mentioned earlier, the direct target of cisplatin is DNA, which can cause DNA cross-linking and double-strand damage. Metabolism may be involved in tumour development because it influences DNA damage repair. The first connection between cell metabolism and DNA repair involves DNA folding (Jeggo and Downs, 2014). Chromatin packaging and remodelling are accomplished through various histone post-translational modifications and DNA modifications, the most common types being methylation (Niculescu and Zeisel, 2002) and acetylation. Most methyl donors are produced during S-adenosylmethionine (SAM) metabolism, which is correlated with methionine, one-carbon metabolism, tetrahydrofolate (THF), and the choline pathway (Niculescu and Zeisel, 2002; Mehrmohamadi et al., 2016). Changes in methionine, THF, and choline concentrations directly affect SAM and thus DNA or histone methylation. The only acetyl donor is acetyl-coenzyme A, which is closely related to TCA (Choudhary et al., 2014; Sivanand et al., 2017). Limiting the number of acetyl donors can destroy normal DNA in affected tissue and influence DNA folding and DNA remodelling (Sivanand et al., 2017). Therefore, regulating methyl and acetyl donors through different metabolic pathways affects the DNA repair process. Second, the availability of metabolites and other nutrients affects the amount and the proportion of nucleotides produced in cells (Rao et al., 2015), thereby affecting DNA repair and replication. Various amino acids are closely related to raw DNA synthesis materials. Glutamine and glycine are involved in purine synthesis (Cory and Cory, 2006; Lane and Fan, 2015), while aspartic acid is related to pyrimidine synthesis. Finally, the regulation of ROS metabolism is also related to DNA damage repair. Strict regulation of cellular oxidation-reduction reaction stress is necessary because high levels of ROS can lead to oxidative stress and oxidative damage to proteins, DNA, and lipids; however, a certain level of ROS is essential to activate signalling pathways involved in multiple biological processes (Shanware et al., 2011; Alleman et al., 2014).

Cells have evolved several ways to balance ROS levels, and GSH is one of the main molecules that scavenge ROS. In addition, studies have found that an important regulator of ROS levels is the transcription factor NRF2, which has been proven to regulate key enzymes of serine metabolism (PHGDH, PSAT1, and ATF4) (DeNicola et al., 2015). In our previous work, we found that reducing the concentration of serine in the medium or inhibiting the activity of PHGDH could reduce levels of H3K4 methylation and promote DNA repair, leading to resistance to cisplatin (X. Zhao et al., 2020). Although tumour metabolic remodelling can affect DNA repair pathways, DNA damage caused by endogenous and exogenous genotoxicity may also lead to cellular metabolic remodelling (Mirzayans et al., 2006). Knowledge of the associations between tumour metabolism and DNA damage and repair is growing, providing opportunities to study the mechanistic basis behind potential metabolic defects in tumours.

## Glycometabolism and Cisplatin Resistance

Glucose is the most demanded nutrient. After ingestion, glucose undergoes glycolysis to produce pyruvate; this can be catalysed to produce lactic acid, which is the final product under hypoxic conditions, or catalysed into acetyl-CoA under normal conditions, ultimately entering the TCA cycle. Tumour cells undergo glycolysis as the main way to generate energy, even under aerobic conditions, owing to the Warburg effect, which is also involved in cisplatin resistance (Warburg et al., 1927). Evidence indicates that increased glucose uptake and enhanced aerobic glycolysis induce intrinsic or acquired resistance to cisplatin in several cancer cell types (X. Y. Zhang et al., 2018b).

In a cisplatin-resistant gastric cancer cell model, glycolysis levels were shown to be significantly increased. Gastric cancer cells were significantly more sensitive to cisplatin after the inhibition of glycolysis via treatment with 2-deoxy-D-glucose, a glucose-competitive substrate (Qian et al., 2017; Varghese et al., 2020), which had the same effect in head and neck cancer cells (Simons et al., 2007). Glucose transporter 1 (GLUT1) is closely related to cell metabolism and is mainly involved in transport of glucose across the membrane to provide energy for cells. Studies have found that inhibiting GLUT1 can improve the sensitivity of oesophageal and head and neck cancer cells to cisplatin (Sawayama et al., 2019). In breast and cervical cancer cells, cisplatin inhibited the expression of GLUT1, GLUT4, LDHA (Manerba et al., 2015), and other glycolysis-related proteins, thereby inhibiting glycolysis.

Alterations in glycolysis affecting cisplatin resistance have been shown to be associated with several enzymes. Enolase1 catalyses the conversion of glycerate-2-phosphate into phosphoenolpyruvate in the ninth step of glycolysis (Qiao et al., 2019). The expression of enolase1 in drug-resistant cells is significantly increased, as proven by proteomic screening. Knocking down enolase1 expression resulted in increased sensitivity of gastric cancer cells to cisplatin (Qian et al., 2017). In this resistance model, higher expression of PDK3 was discovered through gene chip technology; PDK3 functions by preventing pyruvate from being catabolized into acetyl-CoA, which forms a positive feedback loop with HSF1, driving cisplatin resistance (J. Xu et al., 2019). Xu et al. found that cisplatin resistance in ovarian cancer involved higher glucose uptake; moreover, oxidative phosphorylation was modulated by Bcl-2, and targeting Bcl-2 reversed cisplatin resistance by inhibiting glucose metabolism (Y. Xu et al., 2018). M2 pyruvate kinase (PKM2) (X. Wang et al., 2017) catalyses phosphoenolpyruvate to produce pyruvate; in dimer form, it typically supplies energy for tumour cells. In osteosarcoma tumour stem cells, PKM2 is highly expressed and downregulated with metformin, which reduces the uptake of glucose and the production of lactic acid and ATP, thereby increasing cell sensitivity to cisplatin (Shang et al., 2017). The oncogene ALC1 can promote cisplatin resistance in oesophageal cancer cells by activating glycolysis (F. Li et al., 2019). Glycolysis levels increased significantly in cisplatin-resistant T24 cells of bladder cancer, promoting acetate and fatty acid synthesis (Wen et al., 2019).

Inhibition of the glycolytic pathway was shown to increase the sensitivity of drug-resistant ovarian cancer cells to cisplatin (Xintaropoulou et al., 2018). In cisplatin-resistant A549 lung cancer cells, the expression of G6PD (Hong et al., 2018), a key enzyme involved in bypassing the pentose phosphate pathway, was increased, and reducing G6PD could increase cisplatin sensitivity (Hong et al., 2018; Giacomini et al., 2020). Pyruvate participates in oxidative phosphorylation via conversion to acetyl-CoA. Pyruvate dehydrogenase kinase (PDK) can inhibit the conversion of pyruvate to acetyl-CoA (Z. Sun and Xu, 2019; G. Wang et al., 2019). Dichloroacetate (DCA), an inhibitor of PDK, can facilitate the transition from glycolysis to oxidative phosphorylation (Stacpoole et al., 2019; Tataranni and Piccoli, 2019). In drug-resistant ovarian cancer cells, PDK1 expression was significantly upregulated, and knocking down PDK1 expression significantly increased cisplatin sensitivity (Zhang et al., 2019). In cisplatin-resistant head and neck cancer cells, glycolysis enhanced cisplatin resistance and PDK2 expression was increased, whereas DCA reversed this enhancement of resistance (Roh et al., 2016). In addition, microRNAs are involved in the regulation of glucose metabolism during cisplatin resistance. Study have found that the expression of miR-5787 is downregulated in cisplatin-resistant tongue squamous cell carcinoma, promoting the transition from oxidative phosphorylation to aerobic glycolysis; in contrast, high expression of miR-5787 can improve the sensitivity of these cells to cisplatin (W. Chen et al., 2019).

## Amino Acid Metabolism and Cisplatin Resistance

A large number of studies have shown that amino acids are used not only as substrates for protein synthesis but also as metabolites and metabolic regulators to support the growth of cancer cells (Z. Li and Zhang, 2016; L. Sun et al., 2018; G. Wu, 2009). Among the amino acids used in this way, glutamine, serine, and glycine have been widely studied (Nikiforov et al., 2002; Labuschagne et al., 2014). Amino acid uptake and metabolism are abnormal in many cancers that show addiction to specific amino acids. Amino acids promote the survival and proliferation of cancer cells under genotoxicity, oxidative stress, and nutritional stress. Thus, targeting amino acid metabolism is a potential cancer treatment strategy (Wei et al., 2020). Amino acid metabolites and metabolic enzymes also affect cisplatin resistance.

Glutamine, the most abundant amino acid in blood and muscle, maintains the high bioenergy requirements of tumour cells and serves as a precursor for macromolecular biosynthesis (Windmueller and Spaeth, 1974). Glutamine has a pleiotropic role, providing not only carbons but also nitrogen for nucleic acid synthesis. This amino acid can serve as a respiratory substrate that enters the TCA cycle in mitochondria, thereby driving ATP production (Fan et al., 2013). In addition, glutamine supports GSH biosynthesis and NADPH production and is involved in cellular redox homeostasis (Jiang et al., 2019). From the perspective of the current review, the glutamine dependence of cancer cells may represent a metabolic vulnerability of cancer; therefore, enzymes that inhibit the glutamine metabolic pathway could be used in cancer therapy (Obrist et al., 2018). Glutamine intake affects the sensitivity of cells to cisplatin. ASCT2 (SLC1A5) (Liu et al., 2018), a glutamine transporter, was found to be highly expressed in A549 wild-type cells and cisplatin-resistant cells but was negligibly expressed in normal lung fibroblasts (J. Wu et al., 2018). By simulating a polyglutamine delivery system with glutamine macromolecules, SLC1A5 was used to deliver specific therapeutic compounds to glutamine-dependent cancer cells, further sensitizing these cancer cells to cisplatin (C. Wang et al., 2018a). The rapid catabolic metabolism of glutamine mediated by the oncogene KRAS continuously enhances the antioxidant capacity of cisplatin-resistant cells, thus enabling them to tolerate cytotoxicity. However, excessive consumption of glutamine also impedes the growth of cisplatin-resistant cells. Compared with cisplatin-sensitive cells, cisplatin-resistant cells show increased autophagy and are susceptible to glutamine deprivation. In the case of glutamine deficiency, the G1 phase is significantly blocked, and the apoptosis rate is increased (Duan et al., 2018a). Inhibition of glutamine metabolism can increase the sensitivity of drug-resistant ovarian cancer cells to cisplatin (Duan et al., 2018b). Combining the glutaminase inhibitor BPTES with cisplatin significantly increased the apoptosis induction rate of cisplatin-sensitive and cisplatin-resistant ovarian cancer cells (Hudson et al., 2016; Masamha and LaFontaine, 2018).

In addition to glutamine, there are other amino acids that contribute to cisplatin resistance. Cisplatin-resistant lung cancer cells do not primarily use glucose but instead consume amino acids such as glutamine and tryptophan to survive. Compared with cisplatin-sensitive lung cancer cells, IDO1 activity and ROS levels in cisplatin-resistant cells were increased. Inhibition of IDO1 with shRNAs or IDO1 inhibitors increased ROS levels and produced significant growth inhibition only in cisplatin-resistant cells (D. J. M. Nguyen et al., 2020). In our previous work, we found that serine deficiency or insufficiency (12.5, 25, or 50% of serine contained in RPMI-1640 complete medium) during cell culture inhibited the toxicity and pro-apoptotic effects of cisplatin on gastric cancer cells by reducing H3K4 tri-methylation. The addition of serine could reverse the sensitivity of gastric cancer cells to cisplatin (X. Zhao et al., 2020).

## Lipid Metabolism and Cisplatin Resistance

Lipid metabolic reprogramming is a newly recognized hallmark of malignancy. Increased lipid uptake, storage, and fat production in various cancers contribute to rapid tumour growth. Lipids form the basic structure of cell membranes and also function as signalling molecules and energy sources (Cheng et al., 2018; Long et al., 2018). Abnormal lipid metabolism is closely related to the occurrence of cancer (Ameer et al., 2014). Lipids affect cell survival, membrane fluidity and dynamics, and response to chemotherapy; therefore, lipid metabolism is relevant to tumour therapy (Qiu et al., 2015). In recent years, lipid metabolism has also been shown to be closely related to cisplatin resistance. Specifically, metabolic enzymes related to lipid metabolism have an effect on cisplatin resistance. In T24R drug-resistant bladder cancer cells, enzymes involved in acetic acid use (ACSS2) and fatty acid synthesis (ACC) and fatty acid synthesis precursors (acetyl-CoA) levels are increased, leading to higher yields of glucose-derived acetic acid and fatty acids. ACSS2 is highly expressed in cisplatin-resistant tissues, and targeted inhibition of fatty acid synthesis was shown to inhibit bladder cancer cell resistance (Jin et al., 2018). In addition, ω-3 polyunsaturated fatty acids induce apoptosis by ADORA1 and enhance the effect of cisplatin on gastric cancer cells, human lung cancer cells, and melanoma cells (Zajdel et al., 2014; Sheng et al., 2016). In primary HCC cells, carnitine palmitoyltransferase-2 is downregulated to promote adipogenesis of cancer cells, inducing cisplatin resistance of HCC cells and enhancing their oncogenic activity and metastasis potential (Lin et al., 2018). AGPS is a key enzyme in the synthesis of ether-based lipids and is highly expressed in cisplatin-resistant glioma cells (U87MG^DDP^). Reducing AGPS levels can inhibit cell proliferation and increase cisplatin sensitivity (Zhu et al., 2014). FASN is essential for initiating long-chain fatty acid synthesis, which is necessary to meet the ever-increasing demands of cancer cells for membrane, energy, and protein production. FASN is highly expressed in cancer tissues compared with normal fallopian tubes. Bauerschlag et al. found that inhibition of FASN could increase the sensitivity of ovarian cancer cells to cisplatin and induce apoptosis, and reverse cisplatin resistance (Bauerschlag et al., 2015).

## Other Forms of Metabolism and Cisplatin Resistance

In addition to the three major metabolites, other metabolites can affect cisplatin resistance. Vitamin D supplements can reduce the risk of many cancers. Vitamin D sensitizes oral cancer cells to cisplatin and partially reverses cisplatin resistance. Cisplatin enhances the expression of lipocalin 2 (LCN2) by decreasing methylation at the promoter, whereas vitamin D inhibits the expression of LCN2 by increasing methylation and promoting cisplatin chemotherapy (Huang et al., 2019). Vitamin D can also inhibit GPX1; reduce the migration, invasion, and proliferation of oesophageal cancer cells; and reduce cisplatin resistance (Gan et al., 2014).

## Summary and Prospects

Cisplatin is an important tool in the treatment of some solid tumours, including ovarian cancer, testicular cancer, lung cancer, and head and neck cancer. Unfortunately, owing to intrinsic or acquired drug resistance, patients treated with platinum often experience relapses and treatment failures. As discussed in this review, cisplatin-resistant cancer cells have been shown to evade drug toxicity by reprogramming their metabolism. Reprogramming involves all major pathways including cell biosynthesis, energy substrates, redox homeostasis, and signal transduction. Now that the regulatory role of metabolism in cisplatin sensitivity is recognized, further attention should be directed to determining how to reverse tumour resistance to cisplatin by intervening in metabolic processes. As mentioned above, many inhibitors targeting metabolic enzymes combined with cisplatin have synergistic antitumour effects (Table 2). It is noteworthy that an effective combination therapy can be developed by linking the findings of basic research to translational research.

**TABLE 2:** The inhibitor targeting metabolic alterations of cisplatin-resistant tumours.

Metabolic pathway	Target	Inhibitor	Type of study
Glucose metabolism	GLUT1	BAY-876 Sawayama et al. (2019)	*In vitro*
	LDHA	Galloflavin oxamate Manerba et al. (2015)	*In vitro*
	PKM2	Shinkonin Wang et al. (2017)	*In vitro*
	G6PD	6AN, DHEA Hong et al. (2018)	*In vitro*
	PDK	Dichloroacetate (DCA) Tataranni and Piccoli, (2019)	*In vitro*
Amino acid metabolism	ASCT2(SLC1A5)	Resveratrol Liu et al. (2018)	*In vitro*
	Glutaminase	BPTES Masamha and LaFontaine, (2018)	*In vitro*
	IDO1	Epacadostat Nguyen et al. (2020)	*In vitro*
Lipid metabolism	ACSS2	1–2 urea Wen et al. (2019)	*In vitro*
	FASN	C75 Bauerschlag et al. (2015)	*In vitro*

The cost of research and development of inhibitors is high and the cycle is long, which leads to a lack of effective intervention strategies for many of the mechanisms currently recognized; therefore, interventions need to be derived from other perspectives. Nutrients are sources of metabolites and regulating the nutritional status of the body can improve the effect of tumour therapeutics. Diet directly determines the nutritional status of the body; therefore, the effect of regulating diet on tumour metabolism has attracted increasing attention. Many studies have also shown that diet can influence the effectiveness of drugs by altering the metabolic state of tumours. Prevention and blocking of drug resistance through diet control will be a new direction for the further development of cisplatin and other platinum-based treatment strategies. Two aspects of diet are notable: diet affects DNA repair and the rate of tumour cell apoptosis by changing the metabolic state of tumour cells; and diet can reduce the toxicity and side effects of cisplatin by changing the organism’s environment, for example, by co-administering sulphur-containing “chemoprotective agents” with cisplatin (Sooriyaarachchi et al., 2016).

In summary, we have systematically summarized the role and mechanism of tumour metabolism in cisplatin resistance and described prospective drug resistance reversal strategies based on tumour metabolism, thus providing new perspectives for the clinical applications of cisplatin.

## Abbreviations

ACSS2, acyl-CoA synthetase short-chain family member 2; ACL, ATP-citrate lyase; ACC, acetyl-CoA carboxylase; ADORA1, adenosine A1 receptor; AGPS, alkylglycerone phosphate synthase; ALL, acute lymphoblastic leukaemia; ASS1, argininosuccinate synthase 1; ATF, activating transcription factor 4; ATM, ataxia telangiectasia mutated; Bim, BCL2 like 11 belongs to the BCL-2 protein family; BPTES, glutaminase inhibitor; CPT2, carnitine palmitoyltransferase-2; CHEK1, checkpoint kinase 1; CTR1, copper transporter 1; DCA, dichloroacetate; DDR, DNA damage repair; DNA, deoxyribonucleic acid; DNL, *de novo* lipogenesis; FASN fatty acid synthase; FDA, food and drug administration; G6PD, glucose-6-phosphate dehydrogenase; PHGDH, phosphoglycerate dehydrogenase; PSAT1, phosphoserine aminotransferase 1; GPX1, glutathione peroxidase 1; GSH, glutathione; H3K4 tri-methylation di-methyl-histone H3; Lys4 tri-methylation; HR, homologous recombination; HSF1, heat shock transcription factor 1; LCN2, lipocalin 2; MAST1, microtubule-associated serine/threonine kinase 1; MAPK, mitogen-activated protein kinase; MOMP, mitochondrial outer membrane permeabilization; NER, nucleotide excision repair; NHEJ, non-homologous end joining; NRF2, nuclear factor E2-related factor 2; PDK, pyruvate dehydrogenase kinase; PKM2, pyruvate kinase isoenzyme M2; REDOX, oxidation reduction reaction; RNA, ribonucleic acid; ROS, reactive oxygen species; SAM, S-adenosylmethionine; SAT1, spermidine/spermine N1-acetyltransferase; SCD1, stearoyl-coenzyme A desaturase-1; TAGs, triacylglycerols; TCA, tricarboxylic acid cycle; THF, tetrahydrofolate.
